# Nanocomposites and their application in antimicrobial packaging

**DOI:** 10.3389/fchem.2024.1356304

**Published:** 2024-02-26

**Authors:** Adriano Brandelli

**Affiliations:** ^1^ Laboratory of Biochemistry and Applied Microbiology, Department of Food Science, Federal University of Rio Grande do Sul, Porto Alegre, Brazil; ^2^ Center of Nanoscience and Nanotechnology, Federal University of Rio Grande do Sul, Porto Alegre, Brazil

**Keywords:** active packaging, bioactive molecules, food packaging, nanofiber, nanomaterial, nanostructures

## Abstract

The advances in nanocomposites incorporating bioactive substances have the potential to transform the food packaging sector. Different nanofillers have been incorporated into polymeric matrixes to develop nanocomposite materials with improved mechanical, thermal, optical and barrier properties. Nanoclays, nanosilica, carbon nanotubes, nanocellulose, and chitosan/chitin nanoparticles have been successfully included into polymeric films, resulting in packaging materials with advanced characteristics. Nanostructured antimicrobial films have promising applications as active packaging in the food industry. Nanocomposite films containing antimicrobial substances such as essential oils, bacteriocins, antimicrobial enzymes, or metallic nanoparticles have been developed. These active nanocomposites are useful packaging materials to enhance food safety. Nanocomposites are promising materials for use in food packaging applications as practical and safe substitutes to the traditional packaging plastics.

## 1 Introduction

Packaging is the physical entity that functions as the barrier between the contents and the external atmosphere. Packaging materials not only provide containment, protection, convenience and communication, the four basic functions of traditional food packaging, but also create the appropriate physicochemical conditions for the products that are essential to obtain a satisfactory shelf life and maintain quality and food safety during transportation and storage (Rhim et al., 2013; Rydz et al., 2018). Metal, ceramics (glass) and paper (cardboard) are traditional materials for food packaging. These materials are still used, however, the light weight, low price, ease of processing and formability, elasticity in the thermal and mechanical properties, provide plastics advantages for the packaging of food products. The large quantities used for food packaging are the most demanding plastic applications in the form of films, sheets, bottles, cups, tubs and trays (Ncube et al., 2021).

The most used polymers in this sector are petroleum-based materials, such as polypropylene (PP), various grades of polyethylene (PE), polystyrene (PS), polyvinyl chloride (PVC) and polyethylene terephthalate (PET). Although packaging can help to reduce organic waste by preserving food, the substantial increase in the use of synthetic plastics has also posed a number of environmental problems from the waste management point of view (Jadhav et al., 2021; Ncube et al., 2021). As a result, the development and use of biodegradable and/or biologically based materials have received increasing attention from the food industry (Brandelli et al., 2017). The biopolymers or biodegradable plastics are those materials that can be degraded by the action of living organisms, such as bacteria or fungi, to produce final products of the degradation process: CO_2_, H_2_O and biomass under aerobic conditions, or hydrocarbons, methane and biomass under anaerobic conditions (Shaikh et al., 2021). The biodegradable polymers can be classified according to their origin and a variety of raw materials serve as sources for different bio-based polymers (Rydz et al., 2018), which present a diversity of physicochemical and biodegradable properties (Supplementary Table S1).

Meanwhile, several common factors have been identified that limit the industrial application of biopolymers. First, in most cases, their mechanical properties are relatively poor compared to many petroleum-based plastics due to the inherent lower stiffness and strength. Second, many biopolymers are relatively sensitive to water, and some materials dissolve rapidly or show a substantial decrease in mechanical resistance when absorbing water, especially in humid environments. Third, the low level of production, variability among different lots and the high cost restrict its use for a wide range of applications (Perera et al., 2023a; González-Lopez et al., 2023). Some bio-based materials have been developed with the claim to overcome the deficiencies of low heat tolerance that prevented the widespread use of biopolymers. An example is renewable polymers that use ferulic acid and other phenolic antioxidants available in lignocellulosic residues to create functional biodegradable plastic imitators, hydrolyzed in water and fully biodegradable with improved properties (Bascón-Villegas et al., 2022; Blasi et al., 2023). In addition, the use of nanomaterials such as layered inorganic solids has attracted attention by the packaging industry, due to their low cost and substantial enhancements of mechanical and barrier properties (Brandelli et al., 2017).

The use of nanotechnology has been a promise for this sector, since significant improvements can be achieved by the introduction of nanostructures in packaging materials, resulting nanocomposites with improved mechanical, thermal, barrier and functional properties (Taherimehr et al., 2021; Perera et al., 2023a). Moreover, active films developed with nanostructured antimicrobials have the potential for improving safety and quality of packaged foods (Brandelli et al., 2023). In this regard, the main objective of this article is to discuss the potentials of nanotechnology for food packaging purposes, describing the use of nanostructured materials for the development of packaging with antimicrobial properties. Special emphasis is dedicated on incorporation of nanostructures as vehicles for natural antimicrobials in food packaging, including examples of recent studies demonstrating the use of nanostructured materials to improve food safety and shelf-life.

## 2 Nanocomposites in food packaging

**FIGURE 1:**
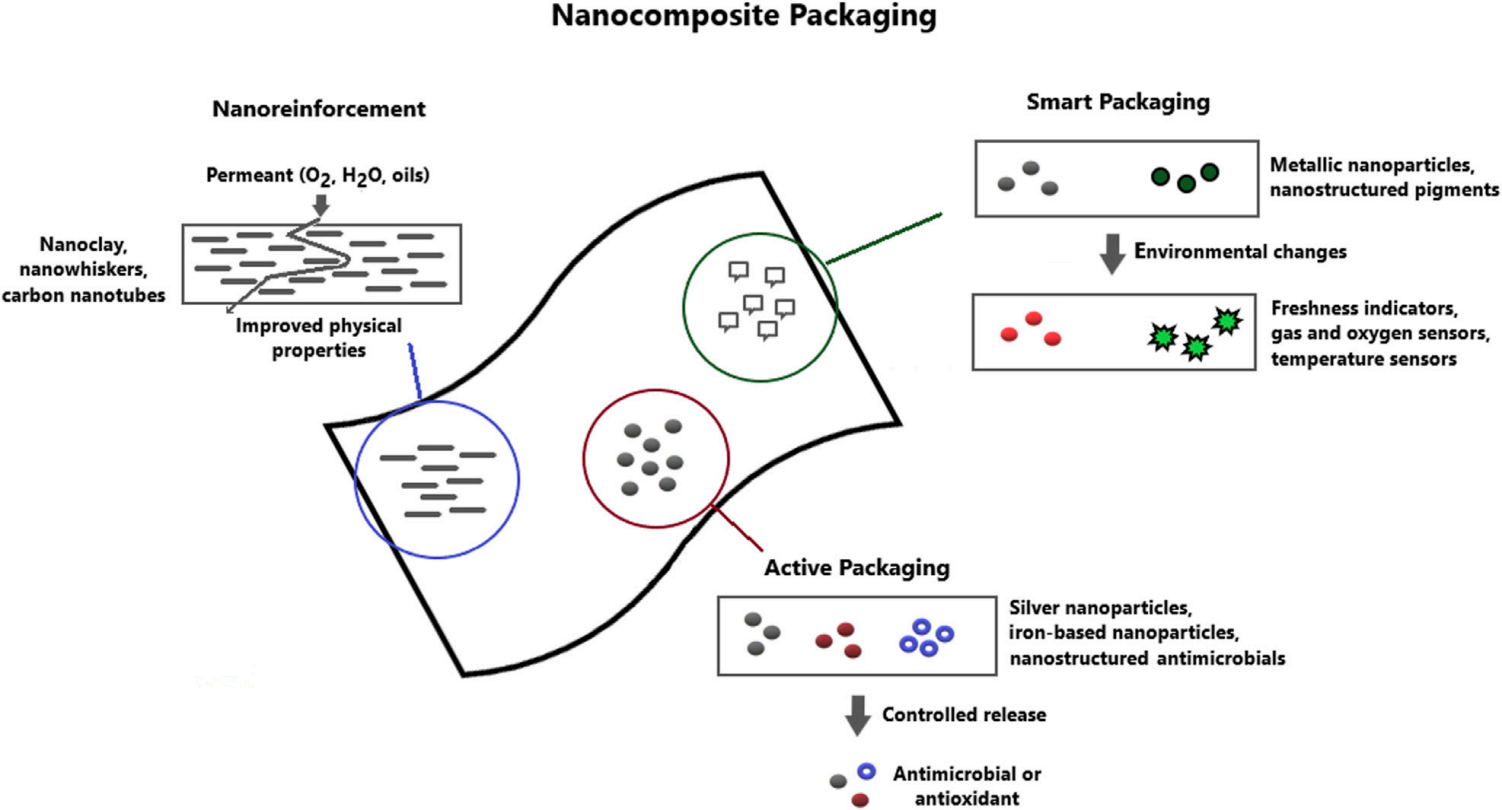
Applications of nanotechnology in the food packaging sector. Nanoclays, nanowhiskers and carbon nanotubes are used for reinforcement purposes improving barrier, mechanical and thermal properties of polymeric films. Active packaging is developed by including metallic nanoparticles and/or nanostructured antimicrobials and antioxidants to provide enhanced shelf life though antimicrobial and oxygen scavenging properties. Intelligent packaging is developed by including nanosensors and nanobarcoding as freshness indicators, gas and oxygen sensors and time-termperature integrators.

In accordance with the market-oriented applications and development trends, three main categories of nanotechnologies can be useful in food packaging applications: nanoreinforcement, nanocomposite active packaging and nanocomposite smart packaging (Figure 1). Nanoreinforcement is used to improve polymer flexibility, gas barrier properties and temperature/humidity stability due to the presence of nanoparticles in polymer matrix materials. Active nanocomposite packages allow interaction with food and the environment and play a dynamic role in food preservation by including antimicrobial systems, oxygen scavengers and immobilized enzymes to fulfill the promises of food protection (Brandelli et al., 2017). Intelligent nanocomposite packaging presents nanodevices in the polymer matrix that can control the state of packaged foods or the surrounding environment and can also act as a protection against fraudulent imitation. Smart packaging is away from the scope of this article and has been widely discussed in recent literature (Brandelli et al., 2020; Azeredo and Correa, 2021; Salgado et al., 2021; Vasuki et al., 2023).

Conventional plastic polymers have been used for the development of nanocomposites for food packaging. Polymers incorporating nanoclays and silver (Ag), zinc oxide (ZnO) and titanium dioxide (TiO_2)_ nanoparticles appear as the most described (Vasile, 2018; Duda-Chodak et al., 2023). These materials are normally prepared with the intention of use in conservation (nanosilver) and barrier (nanoclay) as main applications (Supplementary Table S2). A number of nanocomposites prepared with conventional plastic polymers and nanomaterials in order to improve the properties of the packaging or improve the conservation of the product are described (Sarfraz et al., 2020). When the polymers are combined with nano-reinforcements, the resulting nanocomposites often exhibit significant improvements in thermal and mechanical properties, dimensional stability and resistance to solvents or gases with respect to the pure polymer. Materials that have been used include nanoclays, carbon nanotubes, silica nanoparticles, cellulose or chitin nanowhiskers (Brandelli et al., 2017; Sarfraz et al., 2020). For reinforcement purposes, a good interaction between the matrix and the filling is desired, which is often one of the main challenges faced when developing new nanocomposite materials. It has been observed that the matrix/filler interactions are significantly improved by reducing the size of the reinforcing agent, always taking into account that both phases are compatible and that the filling is dispersed correctly. Nanoclays appear in three different types of morphologies in the polymer/clay nanocomposites (Supplementary Table S3). The exfoliated state that results in a nanocomposite formed by the clay layers delaminated and randomly dispersed in the polymer matrix, results in the best properties of the films due to the optimal interaction between clay and polymer (Rhim et al., 2013; Sarfraz et al., 2020).

Nanoclays have been incorporated in both conventional and bio-based packaging materials. The incorporation of bentonite nanoclay into poly (lactic acid) films prepared by melt processing resulted in a nanocomposite with improved barrier properties against water vapor migration, reducing diffusion by more than 22%. The nanocomposite film showed an extended lifespan and higher ability to sustain elongation in comparison with films without nanoclay filler, suggesting that nanoclay acted as a plasticizer (Oliver-Ortega et al., 2021). In another study, nanocomposite films were prepared combining the antimicrobial properties of chitosan, antioxidant activity of xylan and good mechanical characteristics of cellulose nanowhiskers. The analysis of film microstructure revealed the good miscibility between chitosan/xylan and cellulose nanowhiskers, which improved tensile strength and elongation at break of the nanocomposite. The antibacterial and antioxidant activities were confirmed, indicating the potential application as food packaging material (Bao et al., 2018). Moreover, active cassava starch films prepared with clove essential oil and montmorillonite (MMT) were tested for preservation of fresh strawberries. The incorporation of MMT and essential oil provided better mechanical properties (elongation greater than 180%), improved light barrier performance (opacity greater than 17%) and high antioxidant action due to the occurrence of polyphenols in clove essential oil. Strawberries packed with the active films showed improved parameters of color, acidity and lower microbial contamination during the storage period (Alves-Silva et al., 2022). These examples illustrate the value of nanocomposites in food preservation and their applications in antimicrobial food packaging are detailed in the next sections.

## 3 Antimicrobial food packaging

The initial approaches for the development of active food packaging aiming the inhibition of pathogenic and spoilage microorganisms were based on the direct addition of antimicrobial substances in the polymer matrix. Various synthetic polymers and biopolymers incorporated with antimicrobials have been tested and found effective against susceptible microbial populations (Supplementary Table S4). Several antimicrobial substances, including natural products such as essential oils, antimicrobial peptides, sorbic acid, lactic acid, isothiocyanates, antimicrobial enzymes and modified clay minerals have been employed to develop active antimicrobial packaging materials (Peighambardoust et al., 2021; Popa et al., 2022; Duda-Chodak et al., 2023).

Furthermore, nanostructured antimicrobials have been introduced in food packaging. A series of studies has been dedicated to the addition of Ag nanoparticles into nanocomposite packaging (Table 1). It can be observed that both conventional polymers and biopolymers are good supports for silver nanoparticles of different particle sizes, resulting in inhibition of various microorganisms. Other metals and metal oxides, such as copper (Cu), gold (Au), ZnO and TiO_2_ have the potential to be reduced to nanoparticles for incorporation in antimicrobial food packaging, but are less studied in comparison with Ag nanoparticles (Simbine et al., 2019; Duda-Chodak et al., 2023). In addition, active food packaging materials based on Ag nanoparticles are commercially available. These products are essentially composed by food grade polymers, mostly polypropylene and silicon, incorporating nanosilver as antibacterial agent, with the claim that nanosilver helps to effectively control bacterial growth and maintain product freshness longer (Istiqola and Syafiuddin, 2020).

**TABLE 1:** Use of silver nanoparticles in packaging materials^a^.

Polymer^b^	AgNP (nm)	Antimicrobial property
Polyamide 6	10–20	Inhibition of *E. coli*
PVP	15–25	Reduction of bacterial counts in asparagus
Alginate	5–40	Inhibition of *E. coli* and *S. aureus* in pears
Chitosan	10–25	Inhibition of bacteria (*S. aureus, P. aeruginosa*) and fungi (*A*. *niger*, *C*. *albicans*)
Chitosan/MMT	3–4	Inhibition of *E. coli* and *B. subtilis*
Chitosan/starch	20–25	Inhibition of *E. coli*, *S. aureus*, *B. cereus*
HPMC	40–100	Inhibition of *E. coli* and *S. aureus*
Agar	21.3–23.8	Inhibition of *E. coli* and *L. monocytogenes*
LDPE	10	Reduced bacterial counts in orange juice
LDPE	36	Inhibition of *S. aureus*
LDPE	5.5	Inhibition of *E. coli*, *S. aureus* and *C. albicans*
LDPE	500	Inhibition of molds and total bacteria in berries
PEO	90	Inhibition of *A. acidoterrestris*

^a^ Compiled from Rhim et al. (2013); Simbine et al. (2019); Duda-Chodak et al. (2023).

^b^ LDPE, low density polyethylene; PVP, polyvinyl pirrolydone; HPMC, hydroxyprolyl methyl cellulose; PEO, polyethylene oxide; MMT, montmorillonite.

The European Food Safety Authority (EFSA) evaluated some forms of silver nanoparticles incorporated into food contact materials and their use is approved in food packaging only (EFSA Panel on Food Contact Materials, Enzymes and Processing Aids, 2021). The US Food and Drug Administration (FDA) does not evaluate all products containing nanomaterials or involving the application of nanotechnology, but considers the characteristics of the finished product and the safety of its intended use (FDA, 2014). Although the FDA is not aware of any significant use of nanomaterials in the food packaging market, the Project on Emerging Nanotechnologies (PEN) indicates there is a significant amount of research on engineering nanomaterials intended for food packaging. They point several products already in the US market, such as composite plastic bottles incorporating nanoclays to extend the shelf life of beverages (Bumbudsanpharoke and Ko, 2019; Perera et al., 2023b).

### 3.1 Fabrication of antimicrobial nanocomposites

**FIGURE 2:**
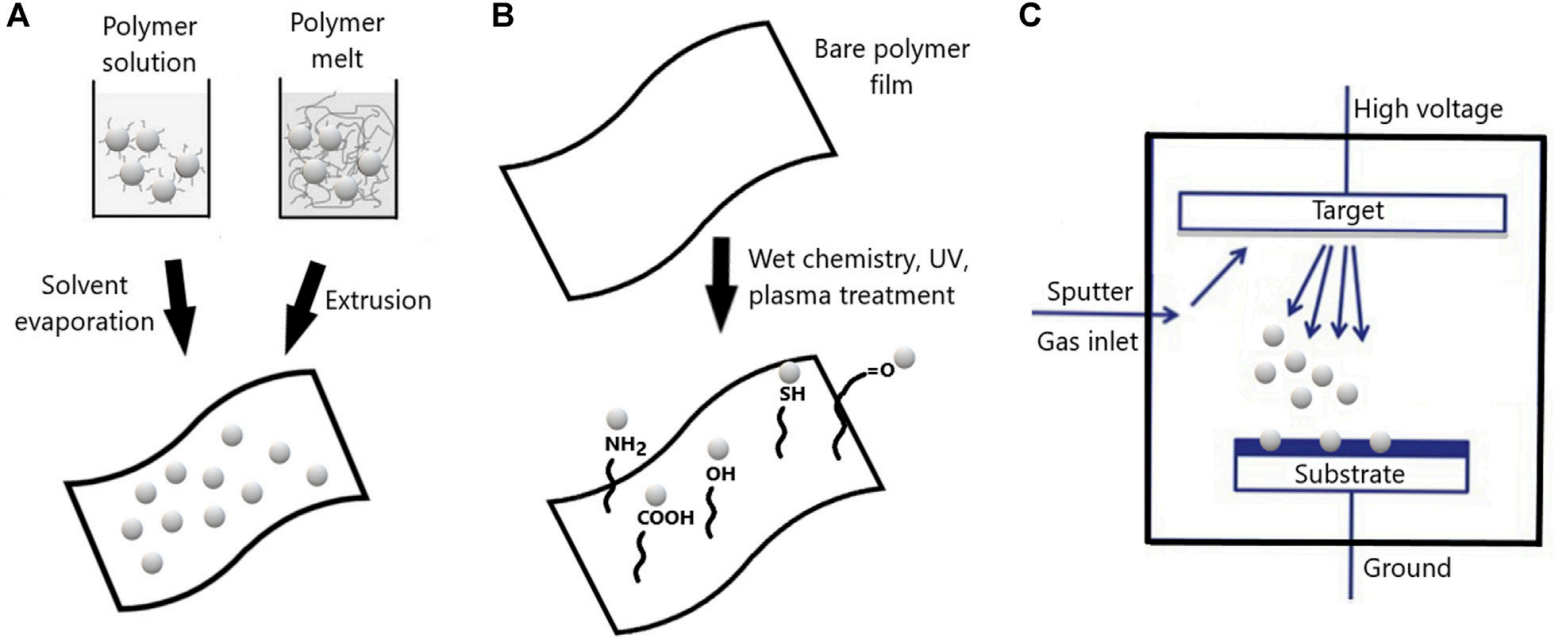
Schematic representation of different strategies for incorporation of antimicrobial nanoparticles into food packaging materials. **(A)** Nanoparticles can be incorporated in polymeric films by casting or thermal methods. **(B)** Grafting of antimicrobial nanoparticles can be achieved after surface modification through pure wet chemistry, irradiation or plasma pretreatment. **(C)** Antimicrobial nanocomposite coatings by plasma deposition.

Diverse strategies can be used to prepare nanocomposite packaging materials presenting antimicrobial activity. Antimicrobial nanoparticles can be incorporated within packaging materials, grafted on packaging surface or deposited as coatings by plasma treatment (Figure 2). The release rate of antimicrobials from nanoparticles is controlled by the diffusion of the substance within the nanoparticle and the external phase, being the chemical potential gradient the fundamental thermodynamic driving force for diffusion. Since diffusional processes in polymers are slow (at least several orders of magnitude below those in liquids) rate controlling physical step in migration is the diffusion of substance from the interior of the polymer to the surface (Dawson et al., 2020). In the case of nanoparticles, the release profile can be manipulated by properties of the encapsulating material and the active substance. The interaction of hydrophobic active substances with lipids in liposomes, for example, may affect the assembly of lipid structure increasing release rate. In nanocomposites prepared with antimicrobial ZnO nanoparticles and CMC, hydrogen bond and electrostatic effect are the main forces that make the nanoparticles absorb to CMC, and slower-release properties are observed in aqueous environments (Ali and El-Molla, 2019).

The incorporation of antimicrobial substances into food packaging is widely used to produce composite active packaging materials. This approach can be achieved by the casting method or thermal processing. The casting process is one of the oldest methodologies for the production of thin polymer films. This method consists of spreading a film-forming solution onto a flat and protected surface, followed by drying and removal of the formed film. The casting method has been extensively used to produce nanocomposite films for various applications, including antimicrobial food packaging. Casting methods have been intensively investigated using different biopolymers, such as starch, chitosan, carboxymethyl cellulose and gelatin, in most cases including silver nanoparticles and nanoclays to produce antimicrobial nanocomposite packaging films (Pal et al., 2021). The thermal processing, such as melt blending, extrusion, and injection molding, has been extensively studied and widely employed for incorporating antimicrobials into polymers. The heat stability of the antimicrobial substance and compatibility with the polymer matrix should be considered in thermal processing. Conventional plastic packaging showing better thermal tolerance, such as LPDE, PP and PLA, are normally employed since processing temperatures are often above 160°C (Gaur et al., 2020). Some natural antimicrobials such as the bacteriocin, nisin have been incorporated into packaging materials by either casting or thermal processes (Popa et al., 2022). However, essential oils are thermal sensitive and the active materials are often prepared by solvent-casting method from water dispersions of biopolymers, such as the nanocellulose films embedded with cinnamon, oregano and thyme essential oils used as active packaging for raspberries (Casalini et al., 2023). The limitation of essential oils for thermal processing can be bypassed by dissolution of polymers in appropriate organic solvents. This strategy allowed the preparation of PBAT films incorporating oregano essential oil. These films with antioxidant and antimicrobial properties reduced *Staphylococcus aureus* and psychrotrophic microorganisms for up to 28 days storage of sliced mozzarella cheese (Cardoso et al., 2022).

Besides incorporating antimicrobial substances into the packaging matrix, nanocomposites can also be immobilized on the packaging surface. In this case, mechanical and thermal properties of the polymer matrix would not be affected, as it comprises a surface treatment. In general, polymer surfaces are intrinsically inert, presenting low surface free energy, poor wettability and poor adhesion capability. Thus, surface treatment is required to overcome this inertia. Grafting of nanoparticles can be achieved by wet chemical pretreatment or irradiation/plasma pretreatment. These pretreatments are realized to generate polar reactive groups on the surface, such as carboxyl, amine, hydroxyl, azide, amidoxime and thiol (Drobota et al., 2022; Purohit et al., 2023). Nonetheless, some polymers have functional groups that make them inherently antimicrobial, such as polyvinylbenzyl ammonium chloride, functionalized polymethacrylates, and *N*-halamine polymers (Wang et al., 2020), cationic polymers containing either quaternary phosphonium salts or quaternary ammonium salts, and natural polymers like chitosan and poly-ε-lysine (Yañez-Macías et al., 2019). A recent study showed that the combination of poly-ε-lysine, chitosan and natamycin could achieve an excellent antimicrobial effect against bacteria and fungi, and decrease the dose of a single preservative. Moreover, high-throughput sequencing revealed that the combination of antimicrobials could reduce diversity and richness of spoilage microbiota in tomato scrambled egg paste, particularly, *Acinetobacter*, *Pseudomonas*, *Aspergillus* and *Fusarium* (Wu et al., 2023).

Antimicrobial nanocomposite materials have also been developed by plasma polymerization, which is a versatile method to render certain functions onto surfaces of a broad range of substrates. The deposition of silver nanoparticles by sputtering has been described. The deposition of silver nanocomposite thin films supported on an organosilicon matrix have been studied in detail by associating plasma polymerization and simultaneous silver sputtering (Izai et al., 2023). Antimicrobial nanocomposite coatings deposited by atmospheric pressure plasma jet process or multilayer deposition, which are mostly composed of three layers where antimicrobial nanoparticles are enclosed between two polymer layers (Kratochvíl et al., 2018; Brobbey et al., 2019).

### 3.2 Nanostructured antimicrobial films

Packaging materials incorporating nanostructured antimicrobial substances have been developed for food application purposes. An interesting area is the preparation of nanocomposite films containing nanostructured essential oils. Alginate films incorporating MMT nanoclay were developed with the addition of essential oils of marjoram, garlic or cinnamon, using Tween 80 as the surfactant. The ingredients were mixed with Turrax, which generates a nanoemulsion of the oils in the polymer/nanoclay matrix. The films caused the inhibition of *Listeria monocytogenes*, *S. aureus* and *Escherichia coli in vitro*. Then, the films containing marjoram oil were tested in wrapping fish fillets and inhibition of *L. monocytogenes* was achieved (Alboofetileh et al., 2016). Edible films of chitosan and whey protein edible films were formulated with the addition of garlic essential oil encapsulated into liposomes. The shelf life of vacuum-packed sausages was evaluated and compared during 50 days of refrigerated storage. The presence of essential oil in the active films retarded the lipid oxidation and growth of major spoilage bacteria, and the best results were obtained for chitosan films containing nanoencapsulated garlic oil (Esmaeili et al., 2020). In another study, active films were prepared by casting through the incorporation of PLA capsules loaded with cinnamon essential oil into biodegradable PBAT films. The discontinuity of polymeric matrix and increased hydrophobicity caused by the addition of PLA and essential oil resulted in decreased values of water vapor permeability. These films showed potential for shelf-life extension of packaged strawberries maintaining fruit quality without dehydration (de Souza et al., 2022). Moreover, extension of shelf life and improved antioxidant activity was observed during chilling storage of meat packed with PVA-curdlan film incorporated with encapsulated thyme essential oil (Zhang et al., 2020). The nanocomposite film prepared with *Zataria multiflora* essential oil incorporated into potato starch/apple pectin/ZrO_2_ nanoparticles greatly increased the stability of packed meat compared with control films without encapsulated essential oil (Sani et al., 2021).

Some major antimicrobial compounds of essential oils, such as terpenes and terpenoids have been used in nanocomposite active films. A gelatin-bacterial cellulose-based nanocomposite incorporating cinnamaldehyde was prepared through Schiff’s base and Michael’s addition reactions of amine groups of gelatin and aldehyde group of cinnamaldehyde. The bacterial cellulose improved structural stability in aqueous environment, enhanced tensile strength and reduced water vapor permeability of the nanocomposite film. The films showed effective antimicrobial activity against *S. aureus* and *E. coli*, and the absence of toxicity against L929 cells suggest the potential application as antimicrobial food packaging (Thongsrikhem et al., 2022). Cinnamaldehyde was also encapsulated into chitosan nanoparticles and embedded into chitosan/PVA/fish gelatin ternary matrix. These bioactive nanocomposites showed antimicrobial activity against foodborne pathogens like *S. aureus*, *L. monocytogenes*, *E. coli* and *Salmonella enteritidis*. In addition, evaluation of storage quality indices, such as pH, TBARS values, color, and microbiological analyses, resulted in 12 days shelf-life extension of rainbow trout fillets wrapped in nanocomposite films (Hosseini et al., 2022). A melt-processed PLA film containing thymol or carvacrol complexed in β-cyclodextrins (β-CDs) was evaluated as a biodegradable active packaging of blackberries and raspberries, using as reference commercial clamshell and control PLA package. After 21 days of storage at 4°C, the berries had high degrees of acceptance, without adverse perception of aroma or flavor for both carvacrol and thymol, and general microbial inhibition was observed for yeast and molds (Velázquez-Contreras et al., 2021). In a recent study, gold nanoparticles and cinnamaldehyde were grafted on paper surface as a strategy to control white film-forming yeasts causing severe spoilage during storage and fermentation of kimchi. The functionalized film with both gold nanoparticles and cinnamaldehyde showed greater antimicrobial activity, since more cinnamaldehyde molecules were adsorbed to the large surface area of nanoparticles (Lee et al., 2023).

**FIGURE 3:**
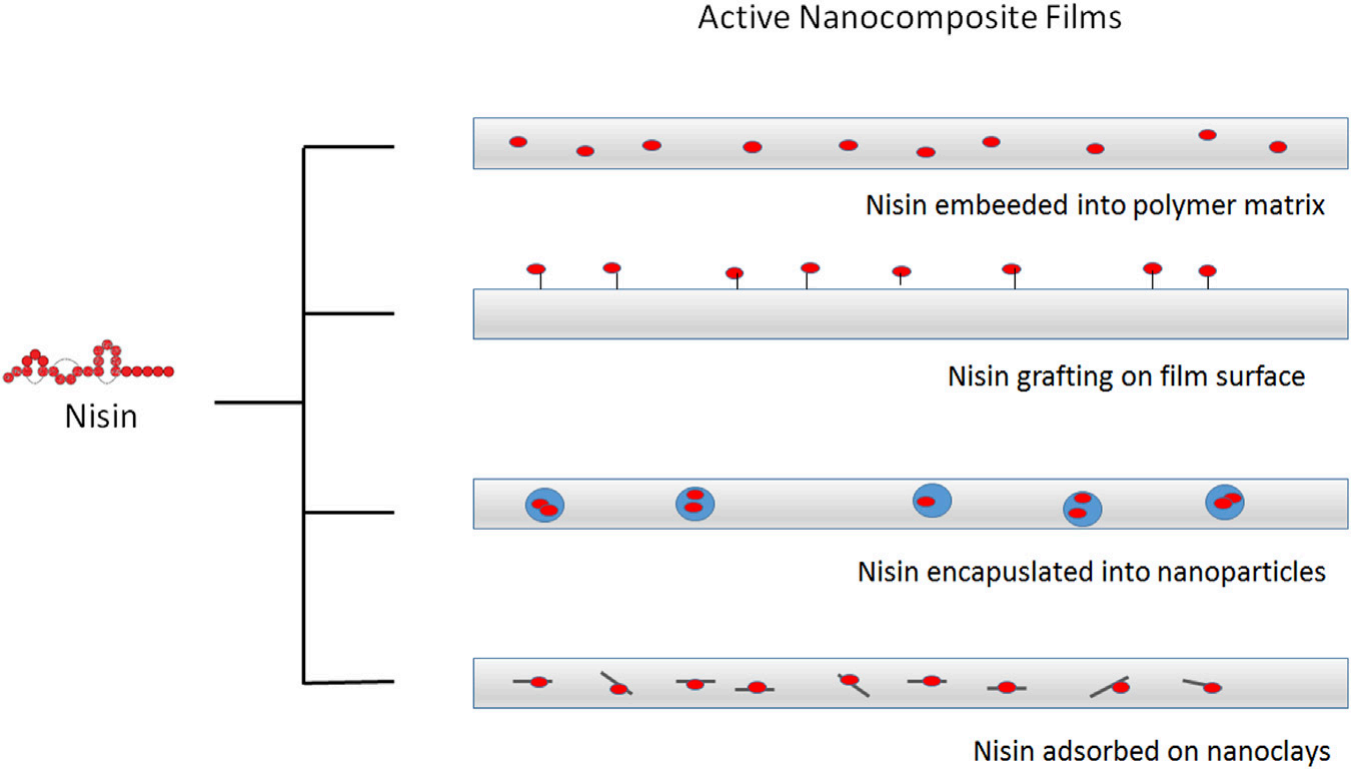
Different strategies for incorporation of nisin into active food packaging. Nisin can be embedded into different polymeric matrixes or grafted on the surface of modified film surface. Nisin can be also encapsulated into nanoparticles or adsorbed onto inorganic nanostructures, such as clays, before incorporation in composite films.

Nanocomposite films incorporating bacteriocins have been described. Antimicrobial activity has been reported for films containing nisin, pediocin and lactacin either alone or combined with nanoclays or metallic nanoparticles (Gumienna and Górna, 2021). Due to the GRAS status and history of safe use in food, different strategies for incorporation of nisin into nanocomposites have been developed. This antimicrobial peptide has been effectively incorporated into nanocomposite films by direct addition, grafting, encapsulated into nanoparticles or adsorbed on nanoclays (Figure 3). As an example, polypropylene-based films were prepared with montmorillonite nanoclay and nisin. The film containing nisin inhibited *L. monocytogenes*, *S. aureus*, *B. cereus* and *Clostridium perfringens* and presented similar mechanical and thermal properties, but increased oxygen permeability when compared with films without nisin. Controlled migration of nisin to media containing Tween 20 or acetic acid was observed. Electron microscopy images of control films and those with 50 g/kg nisin, showed the presence of small pores probably resulting from the separation of the polymer chains due to the presence of nisin (Meira et al., 2014).

**FIGURE 4:**
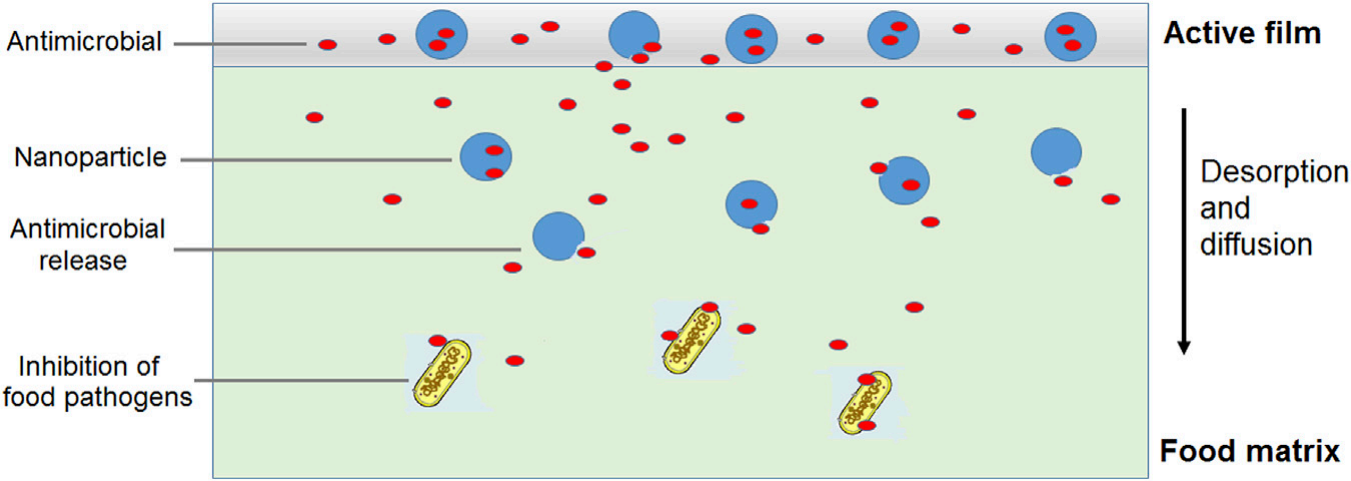
Use of a combination of free and nanoencapsulated antimicrobial substance as active food packaging for extended shelf life.

The incorporation of nanostructured nisin can be a promising method to obtain packaging with prolonged antimicrobial activity. The encapsulated peptide could be released at a controlled rate from the film in contact with the food, resulting in an extended control of undesirable microorganisms. Better antimicrobial effectiveness can be obtained by incorporating a mixture of free nisin and liposome-encapsulated nisin into the polymeric matrix (Imran et al., 2016). The initial burst of a free antimicrobial could significantly reduce the contaminating bacterial populations and subsequently, a sustained release of the encapsulated antimicrobial substance could control the population of remaining bacteria, providing an extended shelf life (Figure 4). In this regard, gelatin and casein films containing nisin-loaded nanoliposomes and halloysite nanoclay as reinforcement were investigated as antimicrobial nanocomposites (Boelter and Brandelli, 2016). Liposomes of about 150 nm in size and with more than 90% encapsulation efficiency for nisin were produced and incorporated to film forming solutions of either gelatin or casein. Halloysite was also included in the formulations at 0.5 and 1.0 g/L and the films were prepared by casting. Inhibition of food pathogens like *L. monocytogenes*, *B. cereus* and *C. perfringens* was achieved with films containing encapsulated nisin, using a milk agar as a food simulating system. However, the antimicrobial activity was impaired with 1.0 g/L halloysite, suggesting that nisin migration was delayed by increased tortuosity of the diffusive path (Perera et al., 2023b).

Moreover, the potential of nisin-loaded rhamnolipid functionalized nanofillers was investigated after embedding in hydroxypropyl-methylcellulose (HPMC) and κ-carrageenan (κ-CR)-based packaging films. When tested on chicken breast filets and cheese slices during cold storage conditions, the active films showed a broad-spectrum antimicrobial activity, reaching up to 4.5 log reduction, and inhibiting the growth of *L. monocytogenes*, *S*. *aureus*, *E*. *coli* and *Pseudomonas aeruginosa* (Niaz et al., 2022).

An innovative approach used corn starch as the polymeric matrix for the development of antimicrobial packaging materials incorporating nisin or pediocin previously adsorbed on halloysite nanoclay. As the halloysite adsorbed with nisin were effective antimicrobial nanostructures, formulations combining different proportions of nisin and halloysite were used for the preparation of films by thermal extrusion. The films showed good mechanical properties and inhibited *L. monocytogenes*, *C. perfringens* and *S. aureus* in milk agar plates. These films were tested as food packaging material using Minas Frescal cheese samples inoculated with *L. monocytogenes* and stored for 15 days. Significant reduction in viable cell counts was observed for all the samples in contact with films containing nisin, and films containing 60 g/kg of nisin resulted in listerial populations below detectable levels (Meira et al., 2016). Following a similar rationale, recent approaches aiming food packaging applications have been developed using cellulose nanocrystals as vehicles for bacteriocins. Effective immobilization of bacteriocins from *Enterococcus faecium* and *Pediococcus acidilactici* onto cellulose nanocrystals resulted in about 50% increase of stability in terms of antibacterial activity (Bagde and Vigneshwaran, 2019). Considering the broad range of antimicrobial activities of these bacteriocins, these nanomaterials may be efficient nanofillers for food packaging applications. The bacteriocin sakacin-A was also conjugated with bacterial nanocellulose, and the active conjugates was tested as a coating mixture applied onto paper sheets. This package material effectively reduce *Listeria* counts in storage trials performed with fresh Italian soft cheese (Rollini et al., 2020).

### 3.3 Nanofibers as antimicrobial packaging material

Nanofibers are very interesting nanostructures for developing high-performance functional packaging materials. Although several methods can be employed to produce polymeric nanofibers, the electrospinning process has received greater attention in active food packaging (Min et al., 2022; Zhang et al., 2023). Electrospun fibers seem more appropriate to encapsulate thermally sensitive substances than fibers prepared by conventional melt spinning or films produced by an extrusion process, or other encapsulation methods. Electrospinning allows the production of unwoven membranes formed by fibers with diameters ranging from tens to hundreds of nanometers that can be used to formulate food packaging materials with improved mechanical properties and very large surface areas (Soares et al., 2018; Wu et al., 2022).

**FIGURE 5:**
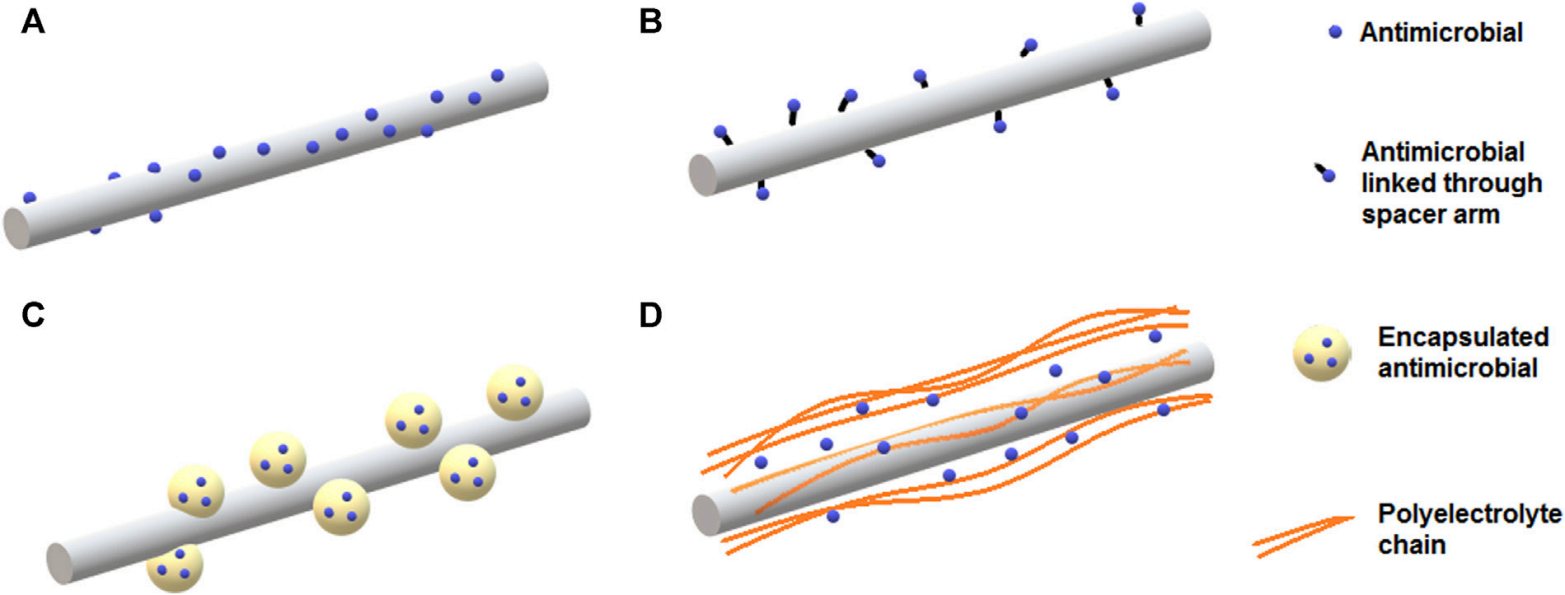
Different strategies to incorporate antimicrobial substances onto nanofibers intended for packaging applications. Antimicrobial substances can be loaded on the surface of nanofibers by **(A)** physical adsorption or **(B)** grafting to surface of functionalized nanofibers. **(C)** Antimicrobial substances encapsulated into liposomes or polymeric nanoparticles can be embedded within or adsorbed onto nanofibers. **(D)** Loading of antimicrobials can be achieved via layer-by-layer assembly often using polyelectrolytes.

Sustainable active packaging can be developed from nanofibers prepared with biopolymers, which can be loaded with large amounts of active substances, such as antimicrobials, antioxidants, enzymes, and reinforcing materials (Zhang et al., 2023). Different strategies can be used to prepare nanofibers as carriers of bioactive compounds. Antimicrobials can be encapsulated into the nanofiber matrix, loaded on the surface by simple physical adsorption or chemically grafted to the surface of functionalized nanofibers. Furthermore, antimicrobials can be encapsulated into nanoparticles and then adsorbed onto nanofibers, or loaded through a layer-by-layer assembly strategy (Figure 5).

Polymeric nanofibers incorporating silver nanoparticles have been developed, with a potential for use in several antimicrobial applications. Silver nanoparticles added to electrospun nanofibers of PLA, PVA, PVP, PEO/chitosan and cellulose acetate show elevated stability and pronounced antimicrobial activity (Zhang et al., 2016). Electrospun nanofibers containing silver nanoparticles have been prepared with a blend of biocompatible polymers such as PCL and gelatin, showing comparable antimicrobial properties, lower cell toxicity, and faster wound closure for mouse skin injury when compared with a commercial silver sulfadizine dressing (He et al., 2021). The electrospinning method was also used for development of silver nanoparticles-PVA nanofibers aiming fruit preservation. The silver-PVA nanofibers displayed antimicrobial activity against food pathogenic bacteria, including *Bacillus cereus*, *S. aureus*, *E. coli*, and *P. aeruginosa*. When used for surface coating of lemon and strawberry, these nanofibers prevented rotting caused by microbial contamination, suggesting their potential use as antimicrobial food packaging (Kowsaila et al., 2019). In another study, nanofiber mats of PVC and cellulose acetate encapsulating silver nanoparticles were fabricated via electrospinning for potential use as active packaging. In a mat-covered-fruit test, strawberries covered by 1% silver-PVC nanofiber mat exhibited the lowest incidence of filamentous fungi at the 7th and 12th day. In agar plates, the electrospun PVC and cellulose acetate nanofibers loaded with silver nanoparticles showed significant growth inhibition of yeasts and filamentous fungi (Tarus et al., 2019).

Graphene oxide nanosheets decorated with silver nanoparticles showed antibiofilm capability against *P. aeruginosa* adhered to stainless steel. Based on this achievement, blended PLGA/chitosan electrospun nanofibers were functionalized with hybrid graphene/silver nanostructures. These functionalized nanofibers were able to inactivate *E. coli*, *P. aeruginosa* and *S. aureus* upon direct contact with bacterial cells (Faria et al., 2015), suggesting an effective utilization to control microbial growth on solid surfaces. Similarly, inhibition of *E. coli* and *S. aureus* was achieved by a multilayer composite prepared with graphene oxide/silver nanoparticles impregnated polyacrylonitrile nanofibers (Sharma et al., 2021).

Naturally occurring antimicrobial substances have been incorporated into nanofibers with the aim of developing packaging materials for improving food safety and quality. Antimicrobial synthetic nanofibers containing plant-derived antimicrobial compounds are often obtained by electrospinning polymers such as PCL, PVA, PEO (Zhang et al., 2017). Some examples of electrospun nanofibers encapsulating antimicrobial phenolics, essential oils, plant extracts, and other natural compounds are presented in Table 2. Other natural substances, such as the bacteriocin nisin, have been also used as antimicrobial agents in nanofiber formulations. For example, nisin was adsorbed to cellulose nanofibers, followed by coating PLA, PP or PE films. The active nanocomposites inhibited *Listeria innocua* and *Brochothrix thermosphacta* on agar plates, and reduced the population of *Listeria* in hamburgers by 1.3 log immediately after contact. An additional reduction of *Listeria* counts (about 1.4 log) was observed after 2 days when the hamburgers were stored in the antimicrobial films (Maresca and Mauriello, 2022). Biodegradable PBAT nanofibers incorporating nisin were produced by electrospinning and their potential as antimicrobial packaging was evaluated (Zehetmeyer et al., 2017). The nanofibers showed a smooth morphology and mean diameter around 800 nm, and the addition of nisin caused no important differences on thermal and mechanical properties. The nanofibers prepared with nisin labeled with a 2-(2′-hydroxyphenyl) benzoxazole fluorochrome revealed that nisin was evenly distributed along the nanofiber. The nisin-loaded PBAT nanofibers inhibited Gram-positive bacteria such as *L. monocytogenes*, *B. cereus* and *C. perfringens*. The electrospinning method was also used for fabrication of nisin-loaded amaranth protein/pullulan nanofibers. Fibers containing encapsulated nisin caused a complete inhibition of *S. thyphimurium* and *Leuconostoc mesenteroides* inoculated in apple juice after 48 h, while *L. monocytogenes* was inhibited by 20 h. Complete inactivation of the same microorganisms was observed for at least 120 h when nisin-loaded nanofibers applied to fresh cheese, suggesting this antimicrobial material can be useful for food applications (Soto et al., 2019).

**TABLE 2:** Examples of natural antimicrobials encapsulated with nanofibers.

Polymer^a^	Antimicrobial	Property	Reference
Chitosan/cellulose	Benzyl isothiocianate	Inhibition of *E. coli*, *S. aureus* and *S. thyphimurium*	Jiang et al. (2022)
PLA/gelatin	Catechin	Inhibition of *E. coli* and *S. aureus*	Ranjbar-Mohammadi and Nouri (2022)
Pullulan/Ethyl cellulose	Cinnamaldehyde	Inhibition of *E. coli* and *S. aureus*	Yang et al. (2020)
PLA/βCD	Quercetin	Inhibition of *S. aureus*, *E. coli* and *K. pneumoniae*	Kost et al. (2020)
PEO/chitosan	Peptide NP10	Inhibition of *E. coli* and *S. aureus*	Yu et al. (2021)
PCL	Natamycin	Inhibition of filamentous fungi in cheese	Veras et al. (2020)
PCL/gelatin	Quercetin	Inhibition of *S. aureus*	Karuppannam et al. (2022)
PLGA	Thymol	Inhibition of bacteria and fungi on fruit	Zhang et al. (2019a)
Starch	Carvacrol	Inhibition of *A. niger* and *Penicillium* sp. in bread	Fonseca et al. (2021)
Zein	Propolis extract	Inhibition of Gram-positive, Gram-negative, *C. albicans*	Moradkhannejhad et al. (2018)

^a^ PLA, poly (lactic acid); PEO, poly ethylene oxide; βCD, β-cyclodextrin; PLGA, poly (lactic-co-glycolic acid); PCL, poly-ε-caprolactone.

The grafting of nisin on the surface of cellulose nanofibers was achieved by surface modification of cellulose nanofibers with 2,2,6,6-tetramethylpyrazine-1-oxyl, which causes an oxidation of glucose residues at C6, resulting in carboxylated cellulose nanofibers. These modified nanocellulose was surface activated with a carbodiimide and then with *N*-hydroxysuccinimide to form a more stable amine ester. The subsequent step was the addition of nisin that was immobilized on nanofiber surface. The nanofiber mats showed antimicrobial activity against *S. aureus* and *Bacillus subtilis* with a substantial 3.5 log reduction (Saini et al., 2016). Electrospinning of zein/PEO solution resulted the formation of uniform fibers, which showed smaller diameter, enhanced mechanical strength and antimicrobial activity by incorporation of nisin (Yu et al., 2023). The zein/PEO nanocomposite mats containing nisin efficiently inhibited the bacterial growth on chicken breast during 12 days storage at 4°C. Therefore, electrospun nanofibers loaded with nisin could be a prospective active packaging for food preservation.

The potential of electrospun poly (ε-caprolactone) nanofibers containing the natural antifungal natamycin was evaluated as a potential packaging material in dairy-based substrates. Large inhibition zones were observed against different fungal strains when skim milk agar was used as a food model. After the controlled release of natamycin was observed in food simulating solutions, the antifungal activity of the nanofibers was evaluated in samples of soft cheese as a real food system. The inhibition of toxigenic strains of *Aspergillus flavus* and *Penicillium citrinum* was evident at the cheese/nanofiber mat interface, suggesting the potential as functional active packaging (Veras et al., 2020). Polyamide nanofibers functionalized with natamycin were prepared by a needleless electrospinning method and evaluated as packaging material. Despite natamycin is a typical antifungal compound, the functionalized nanofibers reduced bacterial growth of *L. monocytogenes*, *S. aureus*, *E. coli* and *Salmonella enterica*, and prolonged the shelf life of chicken breast after 7 days storage at 7°C (Lencova et al., 2022).

## 4 Multilayer and multifunctional packaging

It is often difficult to reach all the desirable properties required for efficient food packaging using a single polymer. This gap can be fixed by the blending of polymers, complex multilayer films, and/or using polymer nanocomposites as food packaging materials. The applications of multilayer nanocomposites for food packaging can be helpful in obtaining materials with improved characteristics (Grzebieniarz et al., 2023). In the case of improved packaging, the incorporation of nanoparticles in the polymer matrix usually enhances the mechanical and gas barrier properties as well as stability of temperature and/or moisture parameters. In active packaging, the incorporation of nanostructured antimicrobials through multilayer packaging strategies may provide the carrier component specific interactions with internal and/or external factors, resulting in increased quality, safety and shelf-life of the packaged food (Brandelli et al., 2017; Sharma et al., 2017).

**FIGURE 6:**
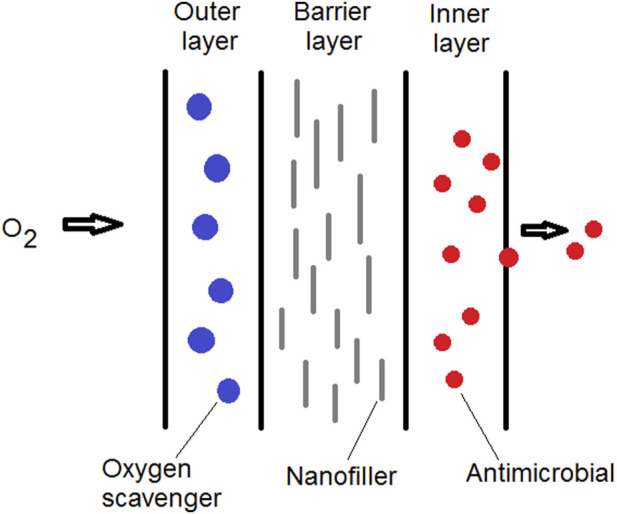
Schematic representation of multilayer packaging film. Different film layers can be incorporated with nanostructures presenting different functionalities.

Anaerobic bacteria may become a major threat when high barrier packaging materials are utilized. Multilayer nanocomposites can be developed for protecting the food against both oxidative and microbial spoilage. For example, one structural layer formed by a polymer/clay nanocomposite with great barrier property can be coupled with other layers with antimicrobial activity and/or oxygen scavenging capability (Figure 6). Polymer/clay nanocomposites with upgraded barrier properties have been used as a barrier layer combined with other structural layers in multilayer packaging materials. Examples of multilayer nanocomposites for rigid food packaging applications include beer bottles, carbonated beverages, and thermoformed containers (Rhim et al., 2013; Anukiruthika et al., 2020). This type of hybrid nanocomposite packaging can be a trend of new flexible packaging materials for the future.

Although some examples of multilayer nanocomposite packaging with improved antimicrobial properties have been described, most developments on multilayer nanocomposite packaging are associated with improved physical or barrier properties (Ganjeh et al., 2024). Multilayer packaging materials can be designed to improve food protection, by adding active ingredients such as antioxidants and antimicrobials. Cinnamon essential oil, for example, was encapsulated within multilayer packaging materials assembled from alginate and chitosan films. The release profile of cinnamon oil was improved as compared with monolayer films, as the multilayer films retained the active ingredients after 10 days storage, while 70% of the oil was lost from the monolayer films (Zhang et al., 2019). In another study, multilayer films consisting of carvacrol-containing LDPE and EVOH were produced by the microlayering process. Carvacrol was melt compounded with LDPE or loaded into halloysite nanotubes prior film production. The multilayered films showed superior oxygen transmission rates and carvacrol diffusivity values in comparison to single-layered carvacrol-containing films produced by conventional cast extrusion. Moreover, the nanocomposite films demonstrated excellent antimicrobial effectiveness in assays using cherry tomatoes as the food model (Krepker et al., 2018). A multilayer packaging material was designed based on a sandwich of electrospun PCL fibers containing quercitin and cellulose nanocrystals between extruded PLA films. Thermal and barrier properties were not significantly improved as compared with 80 μm extruded PLA, probably due to the low thickness of PLC layer, but quercitin provides biological activities to this material (López de Dicastillo et al., 2021).

Multifunctional nanocomposites usually comprise of a mixture of nanostructured materials such as metal nanoparticles, essential oils, bioactive peptides and natural extracts with antimicrobial, antioxidant and other biological functions that are interesting for food contact materials (Vasile, 2018; Grzebieniarz et al., 2023). An active plasticized banana flour nanocomposite film was developed incorporating garlic essential oil as an antioxidant and antimicrobial agent. The nanocomposite was tested as active packaging to maintain the quality of roasted peanuts, in comparison with a commercial plastic packaging of PET/LDPE. The shelf-life estimation of roasted peanuts packaged in both materials based on the peroxide value was similar at high storage temperature (45°C). This new multifunctional nanocomposite showed potential as a packaging material to preserve the quality of oily food products (Orsuwan and Sothornvit, 2018). Another multifunctional nanocomposite for food packaging applications was developed by incorporating titanium dioxide (TiO_2_) and apple peel extract into a PVA/cellulose nanocrystal matrix. The nanocomposite provided an outstanding UV barrier and excellent antioxidant and antimicrobial properties. The multifunctional film effectively protected fresh samples of cherry tomatoes and potatoes from external influences and prolonged their shelf life in food packaging tests (Nguyen and Lee, 2023). Thus, biodegradable and multifunctional nanocomposite films may represent an interesting approach for development of innovative food packaging materials.

## 5 Concluding remarks

Food packaging technology has undergone a wide variety of changes in the last years to become a tunable process for the development of innovative materials with active surface functions. The advances in the strategies for the incorporation of bioactive molecules into nanostructures and the development of nanocomposite packaging materials constitute a topic of great promise in the food sector. Development of novel nanostructured antimicrobials intended for food packaging applications may provide high performance materials that are expected to warrant food safety and quality. The combination of food-grade nanomaterials with effective bioactive compounds can be used for food packaging with enhanced multifunctional properties. Currently, multiple barriers and multifunctional surfaces can meet the complex requirements for a wide range of applications in the food packaging sector. These nanotechnology-based advances represent some of the future directions in the field. Further investigation should address technological solutions for upgrading the stability of natural antimicrobials under some harsh conditions of food processing and storage, and searching for innovative materials and strategies for development of composite packaging suitable for food applications. Assessment of the cost-benefit ratio for the fabrication of nanocomposite packaging materials could be also helpful in the development of cost-effective formulations. Although some studies have addressed the effect of nanostructures on human cell toxicity and gut microbiota, additional understanding is needed on how these very small-sized materials can access human cellular systems and may exert undesirable effects on human health.
