# Supplementary material for: Nanocomposites and their application in antimicrobial packaging
Source: Front Chem. 2024 Feb 26;12:1356304. doi: 10.3389/fchem.2024.1356304 (PMC10925673; doi:10.3389/fchem.2024.1356304)
Supplement: Supplementary file 1 [file DataSheet1.PDF]

Supplementary Material

Frontiers in Chemistry

Nanocomposites and their application in antimicrobial packaging

Adriano Brandelli

Supplementary Table 1. Sources and examples of biodegradable polymers useful for packaging purposes. [a]

Biodegradable polymers	Sources	Examples
Polysaccharides	Agricultural biomass	Cellulose, starch, pectin
	Marine	Chitin, chitosan, alginate
	Microbial	Cellulose, xanthan, curdlan
Proteins	Agriculture	Soy, zein
	Animal waste	Collagen, keratin
Biopolyesters	Microbial	Polyhydroxyalkanoates
	Synthetic	PLA, PCL, PVA, PBAT [b]

[a] Based on Rydz et al. (2018); Shaikh et al. (2021).

[b] PLA, poly(lactic acid); PCL, poly-e-caprolactone; PVA, poly(vinyl alcohol); PBAT, poly(butirate acetate-co-terephtalate).

Supplementary Table 2. Examples of nanostructured nanoclay and nanosilver for improvement of food packaging properties. [a]

Nanomaterial	Polymer	Functionality improvement
Nanoclay	Nylon 6	Barrier (similar to glass)
Nanoclay	Starch	Mechanical, biodegradable
Nanoclay	Chitosan/polylactide	Drug controlled release
Nanoclay	Nylon 66	Rheology
Nanosilver	Polypropylene	Freshness maintenance in fruits
Nanosilver	Copolyester	Antimicrobial activity
Nanosilver	PP, PE PES [b]	Antimicrobial and freshness maintenance

[a] Compiled from Rhim et al. (2013); Guo et al. (2018).

[b] PP, polypropylene; PE, polyethylene, PES, poly(ether sulfone).

Supplementary Table 3. Morphologies of polymer clay composites.

Morphology	Appearance	Characteristics
Traditional phase-separated microcomposite	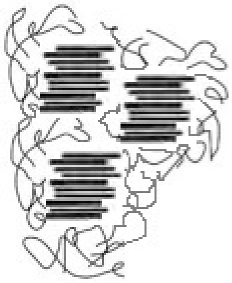	Predominance of tactoids resulting poor physical interaction and poor mechanical properties
Ordered intercalated nanocomposite	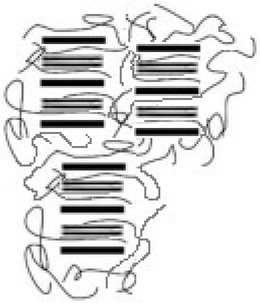	Single polymer chain intercalated in the structure. Less than 20-30Å separation between platelets.
Disordered exfoliated nanocomposite	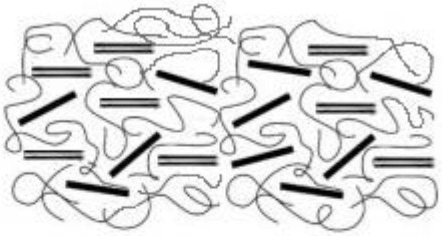	Clay layers dispersed in the continuous polymer matrix. Clay platelets separated by 80-100Å or more.

Supplementary Table 4. Addition of antimicrobial agents in food packaging materials. [a]

Polymer [b]	Antimicrobial	Inhibitory activity
PE, HPMC	Nisin	*B. thermospacta*, *S. aureus*
PLA	Nisin	*L. monocytogenes*, *E. coli* O157:H7
SPI, zein	Nisin, lysozyme, EDTA	*E. coli*, *L. plantarum*
Chitosan	Nisin, ε-polylysine	*S. aureus*, coliforms, yeasts and molds
Alginate	Enterocins A and B	*L. monocytogenes*
PVA, nylon	Lysozyme	Gram-positive bacteria
LDPE, MC/chitosan	Sorbate	Fungi
PE, LDPE	Benzoic acid	Total bacteria
LDPE, nylon	Grapefruit seed extract	Aerobes and coliforms
LDPE	Clove extract	Bacteria and fungi
Alginate	Garlic oil	*E. coli*, *S. aureus*, *B. cereus*
WPI	Garlic oil, oregano oil	Bacteria
SPI	Thyme, oregano oil	*E. coli*, *S. aureus*
Paper	Horseradish extract	*E. coli*
LDPE	Silver zeolite	Bacteria and fungi

[a] Compiled from Popa et al. (2022); Duda-Chodak et al. (2023).

[b] PE, polyethylene; LDPE, low density polyethylene; HPMC, hydroxyprolyl methyl cellulose; SPI, soy protein isolate; PLA, poly(lactic acid); PVA, poly(vinyl alcohol); MC, methyl cellulose, WPI, whey protein isolate.
